# Myeloproliferative Neoplasms: Diseases Mediated by Chronic Activation of Signal Transducer and Activator of Transcription (STAT) Proteins

**DOI:** 10.3390/cancers16020313

**Published:** 2024-01-11

**Authors:** Clifford Liongue, Alister C. Ward

**Affiliations:** 1 School of Medicine, Deakin University, Waurn Ponds, VIC 3216, Australia; c.liongue@deakin.edu.au; 2 Institute for Mental and Physical Health and Clinical Translation, Deakin University, Waurn Ponds, VIC 3216, Australia

**Keywords:** BCR-ABL1, CALR, CML, CNL, CSF3R, ET, JAK2, MPL, MPN, PMF, PV, STAT5

## Abstract

**Simple Summary:**

A group of blood diseases called myeloproliferative neoplasms (MPNs) cause a buildup of certain blood cell types in affected individuals. Each MPN category has a distinct clinical presentation and is associated with mutations in specific genes. However, these various categories all chronically switch on the same cellular pathway, involving signal transducer and activator of transcription (STAT) proteins. This perspective provides new insight for understanding and managing these important diseases.

**Abstract:**

Myeloproliferative neoplasms (MPNs) are hematopoietic diseases characterized by the clonal expansion of single or multiple lineages of differentiated myeloid cells that accumulate in the blood and bone marrow. MPNs are grouped into distinct categories based on key clinical presentations and distinctive mutational hallmarks. These include chronic myeloid leukemia (CML), which is strongly associated with the signature *BCR::ABL1* gene translocation, polycythemia vera (PV), essential thrombocythemia (ET), and primary (idiopathic) myelofibrosis (PMF), typically accompanied by molecular alterations in the *JAK2*, *MPL*, or *CALR* genes. There are also rarer forms such as chronic neutrophilic leukemia (CNL), which involves mutations in the *CSF3R* gene. However, rather than focusing on the differences between these alternate disease categories, this review aims to present a unifying molecular etiology in which these overlapping diseases are best understood as disruptions of normal hematopoietic signaling: specifically, the chronic activation of signaling pathways, particularly involving signal transducer and activator of transcription (STAT) transcription factors, most notably STAT5B, leading to the sustained stimulation of myelopoiesis, which underpins the various disease sequalae.

## 1. Introduction

Myeloproliferative neoplasms (MPNs) represent a family of heterogeneous hematopoietic diseases characterized by the accumulation of single or multiple myeloid cell lineages [1]. This is evident in the blood as well as in the hypercellular bone marrow, frequently leading to bone marrow fibrosis and extramedullary hematopoiesis with progressive splenomegaly [2]. Common symptoms are fatigue, weakness, weight loss, bruising, bleeding, and infections, as well as night sweats and pain in bones and joints [3]. The annual incidence rate across all MPN categories is approximately three cases per 100,000 of the population [4]. However, the prevalence of these diseases is much higher due to the relative longevity of afflicted patients, in part due to the relatively slow progression of symptoms and good responsiveness to a variety of tailored treatments [5].

The World Health Organization (WHO) has classified MPNs into distinct entities, although there is clinical and molecular overlap between them. These categories are chronic myelogenous leukemia (CML), polycythemia vera (PV), essential thrombocythemia (ET), primary (idiopathic) myelofibrosis (PMF), chronic neutrophilic leukemia (CNL), chronic eosinophilic leukemia not otherwise specified (CEL-NOS), and the myeloproliferative neoplasm, unclassifiable (MPN-U) category [1]. ET can progress to PV, and both ET and PV can progress to forms with secondary myelofibrosis (MF), while all MPNs can develop blastic forms [6], with a propensity to progress to acute myeloid leukemia at a collective rate of 5–10% [7]. Amongst these disease manifestations, the myelofibrotic and blastic forms require the most aggressive treatment [6]. This review will provide evidence that, collectively, these diseases can be best explained as perturbations of normal hematopoiesis and the signaling pathways that control this process. Specifically, that due to the acquisition of mutations in hematopoietic stem cells (HSCs) or progenitor cells, a particular signaling pathway becomes chronically activated, leading to the pathological overproduction of particular cell lineages.

## 2. Normal Hematopoiesis and Its Control

**Figure 1:**
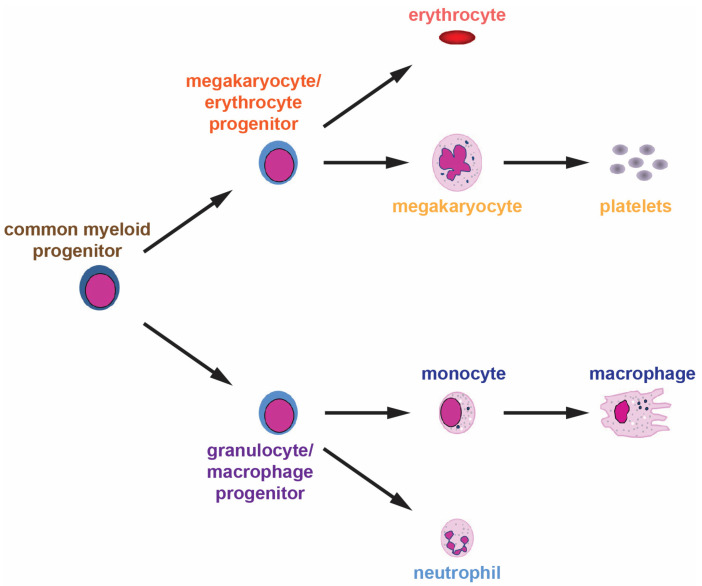
Normal myelopoiesis. The various myeloid cell lineages develop from a common myeloid progenitor through a series of distinct lineage commitment steps with increased differentiation and decreased proliferation as development proceeds.

Hematopoiesis is an ongoing process that generates blood and immune cells from HSCs in the bone marrow throughout their lifespan. These generate daughter cells capable of differentiating into the various cell lineages. For myeloid cells, this occurs via a common myeloid progenitor that can generate either megakaryocyte/erythrocyte progenitors from which platelets and erythrocytes are derived, or granulocyte/macrophage progenitors that yield neutrophils and other granulocytes as well as cells of the monocyte/macrophage lineage (Figure 1).

One important facet of this process is a group of transcription factors that act as intrinsic ‘master regulators’, facilitating the expression of particular gene sets that enable development down specific lineages into fully differentiated cells [8]. For example, the zinc-finger transcription factors GATA1 and KLF1 are essential for promoting erythrocyte development, switching on key genes involved, such as those encoding globins, proteins involved in iron transport, and enzymes in the heme biosynthesis pathway [9]. In comparison, neutrophil development relies on SPI1 and members of the C/EBP family, particularly C/EBPα and C/EBPε, to activate genes encoding key granule proteins and cell surface molecules [10,11]. Finally, FLI-1, NF-E2, and RUNX1 underpin the development of the megakaryocyte/platelet lineage by stimulating the expression of genes encoding platelet granules and other critical proteins [12,13]. These transcription factors also act to repress regulators of other lineages to ensure lineage commitment [14].

**Figure 2:**
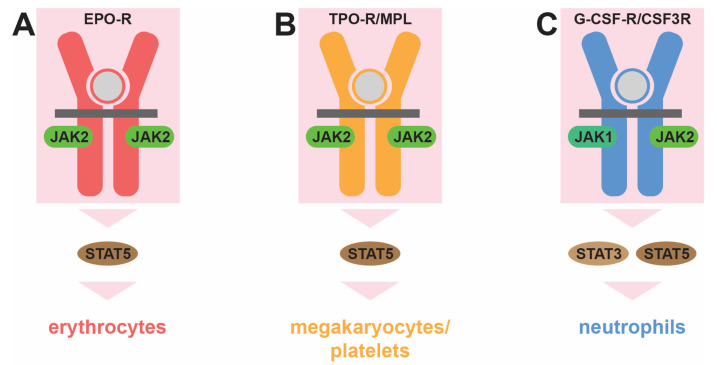
Role of cytokine receptor signaling in myelopoiesis. Distinct cytokine receptors influence the production of specific myeloid cell lineages: the erythropoietin receptor (EPO-R, red) for erythrocytes (**A**), the thrombopoietin receptor (TPO-R/MPL, orange) for megakaryocytes/platelets (**B**), and the granulocyte colony-stimulating factor receptor (G-CSF-R/CSF3R, blue) for neutrophils (**C**). This is mediated by associated Janus kinase (JAK1 and JAK2, green) protein tyrosine kinases and cytoplasmic signal transducer and activator of transcription (STAT3 and STAT5, brown) transcription factors, which are activated transiently when the relevant cytokine (gray) binds to its receptor to influence the production of the indicated myeloid lineage.

However, hematopoiesis is also strongly influenced by a suite of extrinsic factors that modify the cellular output in response to need. The most important of these factors is a set of secreted cytokines that act via corresponding cell-surface cytokine receptors to influence the production of specific cell lineages. Within the myeloid lineages, the erythropoietin receptor (EPO-R) encoded by *EPOR* is critical for the generation of erythrocytes [15], the thrombopoietin receptor (TPO-R) encoded by the *MPL* (myeloproliferative leukemia virus) gene for megakaryocyte/platelet production [16], and the granulocyte colony-stimulating factor receptor (G-CSF-R) encoded by the *CSF3R* (colony-stimulating factor 3 receptor) gene for neutrophil generation [17] (Figure 2). Each of these receptors lacks inherent tyrosine kinase activity and instead utilizes receptor-associated Janus kinases (JAKs), particularly JAK2, to mediate downstream intracellular signal transduction via tyrosine phosphorylation. This process is initiated through the binding of the specific cytokine for each receptor [15,16,17]. This leads to conformational changes that trigger the autoactivation of JAKs, enabling them to phosphorylate other JAKs as well as the cytokine receptor intracellular region on specific tyrosine residues to create docking sites for other signaling molecules. These include the latent cytoplasmic signal transducer and activator of transcription (STAT) proteins, particular the highly related STAT5 proteins, STAT5A and STAT5B, as well as STAT3. These are then in turn activated by tyrosine phosphorylation to form dimers that migrate into the nucleus. Here they facilitate the expression of genes involved in key aspects of this process, including proliferation, survival, lineage commitment, and differentiation, thereby facilitating cytokine-mediated blood and immune cell development, with lineage specificity largely determined by the restricted expression of the respective cytokine receptors [15,16,18]. However, there are differences between STAT proteins in terms of the receptors that activate them and the genes they induce [18], which can also include lineage-specific genes, for example, those involved in iron metabolism in erythrocytes [19].

In each case, the cytokine receptor-mediated responses are tightly controlled. Firstly, there is coordinated cytokine production. For EPO, this occurs in the kidneys in response to oxygen levels, with low oxygen stabilizing hypoxia-inducible factor (HIF) signaling to induce *EPO* gene transcription [15,20]. For TPO, the liver is the key regulatory organ, with aged and senescent platelets triggering expression of the encoding *THPO* gene [21]. For G-CSF, production is stimulated in response to so-called ‘emergency’ signals, such as inflammation or infection, that greatly elevate expression of the cognate *CSF3* gene in various cells [22]. Once produced, these cytokines initiate signaling by their respective receptors, which is separately controlled by a number of mechanisms. These include receptor internalization and trafficking, phosphatases and other constitutive regulators, and also the inducible negative feedback regulators called suppressor of cytokine signaling (SOCS) proteins, to ensure signaling is extinguished in a timely manner [23]. As a consequence, the downstream signal (and the impact on myelopoiesis) normally represents a transient and proportionate response to the cytokine stimulus.

## 3. Underlying Genetic Causes of MPNs

Through the ongoing efforts of many laboratories over the course of several decades, the genetic mutations and variants associated with different MPNs have been thoroughly characterized [24,25,26,27,28] (Table 1). CML is distinctive in having a specific chromosomal aberration called the Philadelphia (Ph) chromosome [24]. The remaining MPNs, collectively referred to as Philadelphia negative (Ph−), typically possess somatic gain-of-function (GOF) mutations in a core set of disease driver genes associated with cytokine receptor signaling [29,30].

### 3.1. BCR::ABL1

CML is associated almost exclusively with a unique acquired chromosomal translocation called [t(9;22)(q34.1;q11.2)]. This results in the fusion of the *BCR* (breakpoint cluster region) gene, located on chromosome 22q11.2, with the *ABL1* (Abelson leukemia 1) proto-oncogene that lies on chromosome 9q34.1. This translocation generates a chimeric *BCR::ABL1* oncogene on the shorter Ph chromosome [24,31], which has become the classical diagnostic hallmark [1]. More than 95% of CML patients express fusion variants of *BCR::ABL1*, with the encoded BCR-ABL1 proteins being constitutively activated tyrosine kinases that drive the leukemic transformation associated with CML via a number of mechanisms [32].

### 3.2. JAK2

Mutations are most common in exon 14 of the *JAK2* gene and result in the substitution of a valine (V) at amino acid 617 to phenylalanine (F) in the encoded JAK2 protein [33]. In addition, various insertions and deletions (indels) occur in exon 12 (ex12) across codons 537–543 in a subset of JAK2 V617F-negative patients [34,35]. The encoded JAK2 V617F and JAK2 ex12 proteins are constitutively active, leading to factor-independent activation of the relevant cytokine receptors [36]. *JAK2* GOF mutations have been collectively identified in nearly all PV patients and over half of ET and PMF patients [28], representing one of the WHO diagnostic criteria for PV, ET, and PMF [30,37].

### 3.3. MPL

Mutations cluster in exon 10 of the *MPL* gene to impact the TPO-R transmembrane domain, most commonly transversion of the tryptophan (W) at amino acid 515 to lysine (K), leucine (L), alanine (A), or arginine (R), or, more rarely, serine (S) at residue 505 to asparagine (N) [38]. Both MPL W515K and W515L have been demonstrated to elicit cytokine-independent responses in relevant cells [39]. *MPL* GOF mutations are collectively present in around 5–7% of ET and 7–10% of PMF patients [28], being a diagnostic criteria for both diseases [30], and are considerably more prevalent in those ET and PMF patients negative for *JAK2* GOF mutations [28].

### 3.4. CALR

This gene encodes an endoplasmic reticulum-associated chaperone protein, called calreticulin, which has been implicated in the trafficking of TPO-R [40]. Heterogenous indels are found in exon 9 of the *CALR* gene, which encodes the C-terminus of calreticulin. Most prevalent are so-called type I (52 bp deletion) mutations followed by type II (5 bp insertion) mutations, both of which lead to a change in reading frame and a truncated protein (p.L367fs*46 and p.K385fs*47, respectively) [41]. These mutations serve to attenuate the binding of calreticulin to calcium, which results in the protein remaining tethered to the TPO-R at the cell surface, thereby resulting in constitutive activation [39,42]. Such *CALR* mutations have been identified in approximately 25–30% of ET cases (preferentially type I mutations) and 20–30% of PMF cases (preferentially type II mutations) and are a diagnostic criterion for both of these diseases [30]. Notably, *CALR* GOF mutations are found predominantly in the Ph− MPN patient cohort that is negative for both *JAK2* GOF and *MPL* GOF mutations [28].

### 3.5. CSF3R

Mutations in the *CSF3R* gene typically impact sequences encoding the transmembrane domain of the encoded G-CSF-R, with the most common being transversion of threonine (T) at amino acid 618 to isoleucine (I) [43], which also results in factor-independent growth [44]. *CSF3R* mutations represent the most predominant genetic lesion in CNL, being found in a majority of patients [45], and serve as a diagnostic factor for this disease [25]. Germline *CSF3R* GOF mutations are also responsible for a hereditary form of CNL [46,47].

### 3.6. Other Mutations/Variants

So-called ‘triple-negative’ (TN) Ph− MPNs, which are found in up to 20% ET and 10–15% PMF patients, lack any of these classical driver gene mutations and instead possess alternative mutations [26,48]. However, non-canonical GOF mutations in *JAK2* and *MPL* have been observed in some of these TN cases, several of which have been shown to lead to constitutive activation [48]. *JAK2* mutations have also been identified in isolated cases of other MPNs, such as in CNL [45] and treatment-resistant CML [32]. Furthermore, loss of heterozygosity has been implicated in increasing the mutant allele burden of GOF mutations in the *JAK2*, *MPL*, and *CALR* genes [48].

Other gene mutations impacting proteins affecting these same cytokine receptor signaling pathways have additionally been identified in patients presenting with alternative MPN-like diseases. For example, hereditary thrombocytosis has been associated with germline GOF mutations in the TPO encoding gene, *THPO*, that result in increased TPO production due to enhanced mRNA translation [49], as well as germline *MPL* GOF and *JAK2* GOF mutations [27]. Hereditary erythrocytosis has instead been linked with germline *EPOR* GOF mutations that lead to truncations of the encoded EPO-R that serve to enhance sensitivity to EPO [50], as well as various other germline mutations (including *EPO* GOF mutations and loss-of-function (LOF) mutations in the *VHL* gene encoding a key regulator) that serve to increase transcription of the *EPO* gene, resulting in high levels of EPO protein [51]. In addition, germline *CSF3R* GOF mutations have additionally been implicated in hereditary neutrophilia [27]. Finally, somatic GOF mutations have also been identified in the *STAT5B* gene, resulting in the transversion of asparagine (N) at position 642 to histidine (H), which causes hyperactivation following cytokine stimulation [52]. These mutations have been demonstrated in patients diagnosed with hypereosinophilic syndrome, with the authors arguing these should be reclassified as chronic eosinophilic leukemia, not otherwise specified (CEL-NOS) [53]. The identical STAT5B N642H mutation has also been found in at least one CNL patient [54].

A number of germline gene mutations/single nucleotide variants (SNVs) that predispose individuals to MPN have also been identified. These include specific alleles of *JAK2* that are associated with an increased risk of developing sporadic MPN [27], including one called 46/1 with a strong predisposition to the acquisition of either JAK2 V617F mutations [55] or TPO-R GOF mutations [56]. Additionally, germline mutations and SNVs have been reported in the *SH2B3* gene, which encodes the SH2B3/LNK adaptor protein that negatively regulates JAK2 signaling downstream of TPO-R and EPO-R, with these LOF mutations associated with ET and PMF [27,57]. A germline *EPOR* GOF mutation that generates an EPO-R P488S variant able to mediate constitutive STAT5 phosphorylation also predisposes individuals to JAK2 V617F-mediated diseases [58].

## 4. Central Role for STAT5

**Figure 3:**
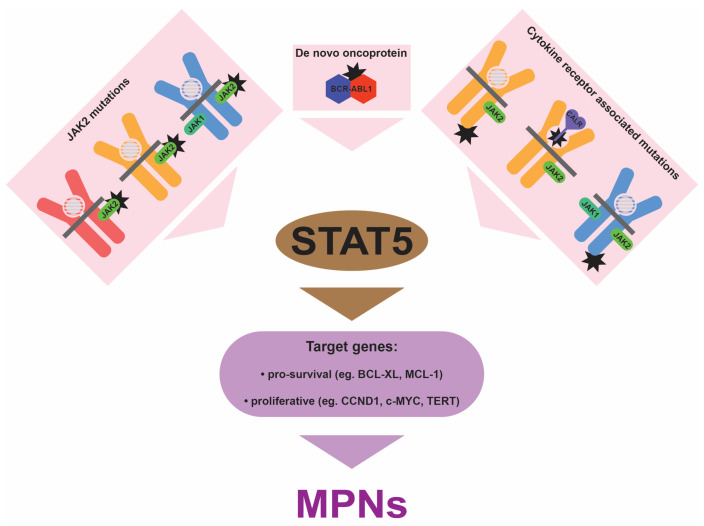
Impact of MPN-associated mutations. Mutations (black stars) can affect the JAK2 protein associated with EPO-R (red), TPO-R/MPL (orange), and G-CSF-R/CSF3R (blue), and calreticulin (CALR) that is associated with TPO-R/MPL, as well as TPO-R/MPL or G-CSF-R/CSF3R themselves, or generate the novel BCR-ABL1 oncoprotein. In each case this leads to chronic activation of STAT proteins, predominantly STAT5. This results in enhanced transcription of a variety of genes that stimulate the excessive myelopoiesis that is associated with all categories of MPNs.

Despite the heterogeneity in the underlying mutations, there is overwhelming evidence that chronic STAT activation, particularly of STAT5 and most notably STAT5B, is central to mediating the impacts of the various mutations at the cellular level (Figure 3). Firstly, chronic STAT5 activation has been consistently observed in MPNs. This includes archetypal *BCR::ABL1*-mediated CML [59], as well as the various Ph− MPNs caused by GOF mutations in *JAK2* [60], *MPL* [61], and *CALR* [41], with chronic STAT3 activation potentially more relevant for GOF mutations in *CSF3R* [62]. Importantly, the chronic STAT activation observed extends to more diverse forms, such as true TN MPNs, where STAT5 has also been shown to be constitutively activated [63]. Secondly, functional studies have demonstrated that STATs are necessary for many of the impacts of these disease driver mutations. Thus, knockdown of the *STAT5* genes abrogated cell survival in cell line models of BCR-ABL1 and JAK2 V617F [64], while ablation of mouse *Stat5* was shown to reduce the severity of MPN induced by either BCR-ABL1 or JAK2 V617F [65,66]. The ablation of *Stat3* resulted in an altered JAK2 V617F phenotype, with decreased neutrophilia, although thrombocytosis was enhanced [67], while the ablation of *Stat1* actually exacerbated JAK2 V617F-induced erythrocytosis in a mouse model despite reducing the thrombocytosis [68]. Amongst the two STAT5 proteins, STAT5B has been demonstrated to be the dominant isoform downstream of BCR-ABL1, being activated to a higher level than STAT5A, with the ablation of mouse *Stat5b*, but not *Stat5a*, able to significantly impact BCR-ABL1-mediated leukemogenesis [69].

A wealth of additional research provides further support for the notion that chronic STAT5 activation is central in MPN pathogenesis. Firstly, the key disease driver gene mutations, *BCR::ABL1*, *JAK2* GOF, *MPL* GOF, and *CALR* GOF, have a strong tendency to be mutually exclusive despite cooperating with an overlapping set of other gene mutations [28], while they all have an impact in the same functional ‘direction’ (that is, to increase STAT5 activation). Both properties represent hallmarks for causative gene mutations that lie in the same pathway. Secondly, several of the common cooperating mutations identified in MPNs intersect with STAT5 at a functional level. For example, CBL is a negative regulator of signaling via its ubiquitin ligase activity, with MPN-associated LOF mutations specifically targeting this activity and shown to be associated with increased cytokine sensitivity and ligand-independent growth [70,71]. SH2B3 is another negative regulatory protein, with the SH2B3 LOF mutations identified in MPNs able to relieve this negative regulation [72]. In addition, the EZH2 protein has been shown to interact with STAT5 to mediate non-canonical transcriptional repression [73], with loss of EZH2 demonstrated to lead to the activation of STAT5-dependent genes in the context of mammary development [74]. Thirdly, STAT5 has also been shown to be important in the subsequent progression of MPNs to cancer, including via targeting p53 [75].

This critical role for STAT proteins, particularly STAT5B, is also reinforced by other observations. Firstly, the pathological consequence of upregulating this pathway shows evolutionary conservation. Thus, a V617F mutation in one of the zebrafish JAK2 paralogues, Jak2a, was demonstrated to replicate many phenotypes of human JAK V617F PV, including enhanced activation of the zebrafish STAT5B homologue Stat5.1 [76]. Furthermore, expression of Stat5.1 GOF mutants caused the expansion of zebrafish myeloid cell populations [77]. Similarly, an activating mutation in the single fruit-fly JAK named Hopscotch resulted in a leukemia-like disease that was dependent on its sole STAT protein, called Marelle [78,79]. Secondly, a similar paradigm is emerging in other hematological malignancies. For example, a suite of mutations that increase the flux through the IL-7R/JAK1/JAK3/STAT5 pathway have been reported in acute lymphoblastic leukemia (ALL) [80]. In addition, in large granular lymphocyte leukemia (LGL-L), a chronic proliferative disorder of mature T and NK cells, the most common GOF mutations are in the *STAT3* and *STAT5* genes, with the latter associated with the most aggressive forms [81]. This contrasts with other hematological malignancies, such as peripheral T-cell lymphomas, particularly the anaplastic large cell lymphoma (ALCL) form of the disease, where GOF mutations in STAT3, but not in STAT5B, have been observed [82]. Finally, a preeminent role for STAT5B over STAT5A has been broadly observed across several hematological malignancies [52].

## 5. Implications for the Clinic

The central role of chronic STAT activation in MPNs, particularly of STAT5, makes the restoration of signaling to non-pathological levels an attractive goal for therapeutic intervention. Indeed, pharmacological agents that have displayed efficacy against relevant MPN-associated mutants have almost uniformly demonstrated a concomitant significant reduction in STAT5 activation (Table 2). Thus, CML is treated with a variety of tyrosine kinase inhibitors (TKIs) specific for the ABL1 kinase, from the first generation imatinib to the third generation ponatinib that targets the imatinib-resistant T315I variant, which all significantly reduce STAT5 activation [3]. Similarly, a series of TKIs with varying degrees of specificity for JAK2 have been developed for Ph− MPNs; these also significantly blunt STAT5 activation. They include, for example, Ruxolitinib, which is employed clinically as a first-line therapy in myelofibrosis and as a second-line therapy in PV [83]. Alternative agents remain in development, including other JAK2 selective inhibitors such as WWQ-131 [84], as well as LCP4, a 20-residue cyclical peptide antagonist of TPO-R shown to be effective in inhibiting the effects of TPO in cells from MF patients [85].

However, STAT5B and, to a lesser extent, STAT3 clearly represent attractive therapeutic targets themselves in MPNs, with specific inhibitors being a high priority. A range of these are in various stages of development, including those targeting activation, DNA binding, dimerization, or translation; these are typically peptides/peptidomimetics, small molecules, and oligonucleotides [86]. Several small-molecule SH2 domain inhibitors have been identified for STAT5. Of these, AC-4-130 was able to decrease STAT5 activation and transcription, as well as proliferation and clonogenic growth in AML cell lines and MPN stem cells [87,88]. Similarly, the compound 17f was also able to inhibit phosphorylation of STAT5 (but not STAT3) and growth in both CML and AML cell lines [89], while ISST5-002 prevented STAT5 phosphorylation and dimerization in response to JAK2 V617F and BCR-ABL1 [90]. The STAT5 protein duplication presents a challenge for specific inhibitor development due to the high conservation of STAT5A and STAT5B. However, compounds such as Stafib-2 have been demonstrated to selectively bind to the SH2 domain of STAT5B (but not STAT5A), with a pro-drug version able to selectively inhibit STAT5B phosphorylation and induce apoptosis in BCR-ABL1-positive K562 cells [91]. A range of small-molecule inhibitors have also been developed for STAT3 [86], with one of these, LLL-3, able to repress BCR-ABL1-positive cell proliferation and synergize with imatinib [92]. Alternatively, STAT DNA binding can be targeted with multiple strategies. Thus, a decoy STAT5 oligonucleotide was able to downregulate the expression of key STAT5 target genes and growth in the K562 model of BCR-ABL1-mediated CML by sequestering activated STAT5 proteins [93]. Alternatively, a peptide aptamer targeting the STAT5 DBD was able to suppress target-gene expression and viability in K562 cells [64]. A range of STAT3 DNA binding inhibitors have also been developed [94,95]; these may also be relevant to certain MPN patients.

However, it is important to acknowledge that a number of other less sophisticated treatment regimens remain very useful clinically, including phlebotomy and cytotoxics, such as hydroxyurea and interferon α, to reduce hematopoietic cell mass, with low dose aspirin employed to provide protection against thrombotic events [83]. In addition, it is clear that other pathways are also involved in MPNs, including PI3K/mTOR, IGF1R/IRS, and MAPK, which can remain activated in the presence of JAK2 inhibitors [96]. The IGF1R/IRS inhibitor NT157 was able to decrease JAK2/STAT signaling, proliferation, and viability in JAK2 V617F-positive cell lines [97], while PI3K pathway inhibitors synergized with JAK2 inhibitors in MPN [96]. There are also alternative approaches that are more indirect, such as interferon alpha, which can elicit partial deep remission especially in JAK2 V617F-mediated disease through the activation of anti-proliferative/pro-apoptotic responses via STAT1 [98].

## 6. Conclusions

MPNs represent significant heterogeneous diseases associated with a variety of symptoms and underlying gene mutations/variants, both of which overlap. However, the mutations and variants functionally coalesce to mediate the chronic activation of signaling, predominantly of the STAT5 transcription factor, which underpins the excessive production of myeloid cells and lies at the core of each of these disorders. This understanding of the central role played by chronic STAT5 activation provides a useful lens to view the various clinical manifestations of MPNs, including the overlapping nature of different categories of MPNs, and, importantly, the approaches that may provide therapeutic benefit. The strong conservation of this pathophysiology means that relevant animal models offer significant opportunities to further investigate these diseases and their treatment.

## Figures and Tables

**Table 1:** Genetic changes associated with MPNs.

MPN Category	Genetic Change	Frequency
CML	*BCR::ABL1*	>95%
PV	*JAK2* GOF	~98%
ET	*JAK2* GOF	55%
	*MPL* GOF	5–7%
	*CALR* GOF	25–30%
PMF	*JAK2* GOF	60%
	*MPL* GOF	7–10%
	*CALR* GOF	20–30%
Hereditary thrombocytosis	*THPO* GOF	nd
	*JAK2* GOF	nd
	*MPL* GOF	nd
Hereditary erythrocytosis	*EPOR* GOF	nd
	*EPO* GOF	nd
	*VHL* LOF	nd
CNL	*CSF3R* GOF	>80%
Hypereosinophilic syndrome	*STAT5B* GOF	nd

Abbreviations: CML: chronic myelogenous leukemia; CNL: chronic neutrophilic leukemia; ET: essential thrombocythemia; GOF: gain-of-function; LOF: loss-of-function; MPN: myeloproliferative neoplasm; nd: not determined; PMF: primary myelofibrosis; PV: polycythemia vera.

**Table 2:** Approved MPN treatments.

Disease	Treatment	Effect	Application
CML	imatinib, dasatinib, nilotinib, bosutinib, ponatinib	ABL1-selective inhibitors	1st line therapy
PV	hydroxyurea, phlebotomy	cytoreductive	1st line therapy
	aspirin (low dose)	antithrombotic	1st line therapy
	ruxolitinib	JAK2-selective inhibitors	2nd line therapy
MF	ruxolitinib, fedratinib, pacritinib	JAK2-selective inhibitors	1st line therapy
Various MPNs	interferon α	antiproliferative, pro-apoptotic	alternative therapy
